# Effect of maternal beta-blocker treatment on mean fetal heart rate

**DOI:** 10.1016/j.xagr.2024.100423

**Published:** 2024-11-20

**Authors:** Sarah Hautier, Thi Minh Thu Nguyen, Arane Kim, Tiphaine Barral, Dominique Luton

**Affiliations:** 1 Assistance Publique-Hôpitaux de Paris, Service de Gynécologie-Obstétrique, Hôpital Bicêtre, Université Paris Saclay, Le Kremlin-Bicetre, France (Hautier and Luton); 2 Assistance Publique-Hôpitaux de Paris, Service de Gynécologie-Obstétrique, Hôpital Bichat, Paris, France (Nguyen and Barral); 3 Centre Hospitalier Universitaire Pellegrin—Service de Gynécologie-Obstétrique, Bordeaux, France (Kim)

**Keywords:** beta-blocker, bisoprolol, mean fetal heart rate

## Abstract

**Background:**

During pregnancy, the prescription of beta-blockers to the mother may be necessary for pre-existing chronic conditions. Their use raises concerns due to potential effects on the fetus.

**Objectives:**

This study aimed to investigate the impact of beta-blockers on mean fetal heart rate in pregnant women treated with these medications compared to an untreated patient group.

**Study Design:**

This was a retrospective case-control study involving 90 patients, divided into two groups: 45 patients on beta-blockers and 45 untreated patients. Included patients delivered singleton pregnancies after 24 weeks of gestational age at two university hospitals in Île-de-France between 2009 and 2021. They were matched based on age, parity, and gestational age at delivery. Fetal heart rate and maternal heart rate were recorded on the day of delivery. Pregnancy outcomes were studied secondarily.

**Results:**

There was no significant difference in mean fetal heart rate between the two groups: 87% of fetuses from mothers treated with beta-blockers had a heart rate between 110 and 150 bpm, compared to 93% of fetuses in the second group (*P*=.71). Among patients taking beta-blockers, the most commonly used treatment was bisoprolol.

**Conclusion:**

The study did not reveal a significant effect of beta-blockers on fetal heart rate. However, close monitoring and appropriate clinical management are still necessary for pregnant patients on beta-blocker treatment due to other potential implications like intra-uterine growth restriction for both the mother and the fetus.

**AJOG Global Reports at a Glance**

**Why was this study conducted?**

This study aimed to assess the impact of beta-blockers on fetal heart rate in women treated during pregnancy.

**What are the key findings?**

IThe main findings indicate no significant difference in fetal heart rate between women treated with beta-blockers and untreated women.

**What does this study add to what is already known?**

This original study contributes to the literature by providing specific data on the effects of beta-blockers on fetal heart rate, emphasizing the importance of routine fetal monitoring interpretation in treated patients.

## Introduction

Beta-blockers are widely used in the treatment of various conditions, including hypertension,^1^ cardiac arrhythmias,^2^ migraines,^3^ and certain cardiovascular diseases such as Marfan syndrome.^4^ Their prescription may also be necessary during pregnancy, primarily for pre-existing chronic conditions.

Prescribing beta-blocker treatments during pregnancy raises concerns due to their potential effects on both the mother and the fetus. Indeed, beta-blockers can interfere with the physiological adjustments necessary for pregnancy: increased cardiac output, heart rate, and blood volume, and decreased peripheral vascular resistance.^5^ Previous studies have suggested several potential adverse effects associated with the use of beta-blockers during pregnancy. These adverse effects include increased risks of intrauterine growth restriction,^6^ neonatal hypoglycemia,^7^ neonatal bradycardia,^7^ hypotension,^8^ and prolonged neonatal hospitalization.^9^

Due to their inhibitory action on beta-adrenergic receptors, beta-blocker treatment can lead to a decrease in maternal heart rate. This hemodynamic change in the mother raises questions about the treatment's impact on fetal heart rate.^10^ An in vivo study in mice showed that administration of propranolol resulted in a decrease in fetal heart rate associated with an increase in heart rate variability, whereas saline had no effect on either mothers or fetuses.^11^ These results, which seem contradictory at first glance, testify to the lack of knowledge about beta-blocker pharmacology, particularly during pregnancy. Any potential alteration in fetal heart rate in humans could have significant implications for obstetric monitoring and particularly for the interpretation of fetal heart rate monitoring. Indeed, a significant variation in fetal heart rate can be an indicator of fetal distress, requiring rapid medical intervention to prevent serious complications such as fetal asphyxia and hypoxia.^12^ Due to the underlying maternal conditions for which beta-blockers are prescribed, there is an increased risk of fetal distress. In this context, accurate interpretation of fetal heart rate monitoring becomes even more crucial.

This study aims to determine whether the use of beta-blockers during pregnancy alters the mean fetal heart rate among patients treated with beta-blockers, compared to fetuses whose mothers receive no treatment.

## Methods

For this study, we conducted a retrospective case-control study involving 90 patients, divided into two groups of 45 patients each. The first group consisted of pregnant patients treated with beta-blockers, while the second group was comprised of pregnant women receiving no treatment.

The data collected included all patients on beta-blockers who gave birth after 24 weeks of gestation to a singleton pregnancy in two hospital-university maternity units in Ile de France, specialized in the management of maternal pathologies, particularly cardiac, between 2009 and 2021. Data were collected from the patients’ computerized medical records and anonymized.

Between the two groups, patients were matched based on age, parity, and gestational age at delivery to minimize potential biases associated with these factors. The control patient was the first patient corresponding to these matching criteria in our database and not already included in the study.

Patients on Labetalol only and those treated with Propranolol for Graves’ disease were excluded from the analysis, as the mechanism of action of Labetalol (a noncardio-selective alpha/beta-blocker) and the impact of Graves’ disease on maternal and fetal heart rates^13^ could introduce biases into our results.

The demographic characteristics (age, parity, and body mass index) of the patients are presented in Table 1. Information regarding the type of beta-blocker treatment, its indication, and dosage were extracted from medical records and presented in Table 2. In case of changes in dosage during pregnancy, the highest dosage was retained.

**Table 1:** Main characteristics of untreated patients and patients with beta-blockers

	Untreated patients *n*=45 (%)	Patients with beta-blockers *n*=45 (%)	*P*
Age, median (IQR)	31 (27–34)	31 (27–34)	1
Parity			
I parous	25 (56)	25 (56)	1
II parous	18 (40)	18 (40)	
III parous	2 (4.4)	2 (4.4)	
BMI (kg/m^2^)			
<18.5	2 (4.4)	9 (20)	0.11
18.5–24.9	23 (51)	24 (53)	
25–29.9	11 (24)	5 (11)	
30–34.9	5 (11)	2 (4.4)	
35–39.9	0 (0)	1 (2.2)	
>40	2 (4.4)	1 (2.2)

**Table 2:** Details of treatment received by patients

Treatment (INN)	*n* (%)	Daily dose mg	Indication
Atenolol	14 (31)	50–100 mg	Marfan syndrome^14^
Bisoprolol	21 (46)	2.5–10	Marfan syndrome^18^ Others^3^
Labetalol	1 (2)	200	Marfan syndrome
Nebivolol	1 (2)	10	Marfan syndrome
Propranolol	8 (17)	40–120 mg	Marfan syndrome^2^ Others^6^

The evaluation of mean fetal heart rate was performed by reading the fetal heart rate monitoring and manually assessing the baseline heart rate on the day of delivery, during labor, or before scheduled C-section, depending on the obstetric context. Situations of suspected intrauterine infection or maternal fever were excluded due to their potential influence on fetal heart rate. It was classified into three categories: less than 110 bpm, between 110 and 150 bpm, and greater than 150 bpm, following the usual definition of normal fetal heart rate.^14^

Maternal heart rate on the day of delivery was also recorded from the fetal monitoring recordings or surveillance sheets in the delivery room or operating room. This rate was categorized into three categories for statistical comparison: less than 60 bpm, between 60 and 100 bpm, and greater than 100 bpm.

Regarding obstetric data, gestational age and mode of delivery were recorded. For neonatal data, fetal weight, and birth percentile according to Audipog curves^15^ were noted.

A descriptive analysis was conducted to describe the characteristics of the study population. Quantitative variables are expressed as mean with confidence interval or median with interquartile range, while qualitative variables are expressed as number and percentage. Comparison of qualitative variables was performed using a Chi-squared test or Fisher's exact test when assumptions were not met, and means were compared using Student's *t* test. An alpha risk of 5% and a significance level of 0.05 were considered in all analyses. Data were processed using R software via the *P* value interface.^16^

The study was approved by an ethics committee (2024-OBS-0601).^17^

## Results

In terms of demographic characteristics, there was no significant difference in age between the two groups, with a median age of 31 years. As shown in Table 1, the two groups were also similar in terms of parity and body mass index.

For the treatment received by patients on beta-blockers, as depicted in Table 2, a large majority were treated for Marfan syndrome, accounting for 80% of the cohort (*n*=36/45). Other indications for treatment included sinus tachycardia (*n*=2), migraine pathology (*n*=2), and cardiomyopathy (*n*=2). One patient was treated due to dilated cardiomegaly, another for rheumatic mitral insufficiency, and the last for Bouveret-Hoffmann syndrome.

Bisoprolol was the most frequently prescribed treatment (*n*=21, 46%), at doses ranging from 2.5 to 10 mg per day, followed by atenolol, mainly for Marfan syndrome. Notably, the majority of patients, 37/45 (82%), were already on beta-blocker treatment before pregnancy. Two patients (4.4%) started beta-blockers during the first trimester of pregnancy, two (4.4%) during the second trimester, and 4 (8.8%) during the third trimester. One patient received bisoprolol followed by labetalol due to poor tolerance.

The main analysis of the study, presented in Table 3, revealed no significant difference in the distribution of mean fetal heart rate between the two groups across the three categories mentioned earlier. Most fetuses, regardless of maternal beta-blocker use, had heart rates between 110 and 150 beats per minute, with similar percentages in both groups (87% vs 93%, respectively), *P*=.71. These findings suggest that beta-blocker use does not significantly impact the average fetal heart rate.

**Table 3:** Maternal and mean fetal heart rate the day of delivery among untreated patients and patients with beta-blockers

Colonne1	Untreated patients *n*=45 (%)	Patients with beta-blockers *n*=45 (%)	*P*
Maternal heart rate			
<60/min	1 (2.2)	7 (18)	<.01
60–100/min	37 (82)	30 (79)	
>100/min	7 (16)	1 (2.6)	
Mean fetal heart rate			
<110/min	2 (4.4)	0 (0)	.71
110–150/min	39 (87)	27 (93)	
>150/min	4 (8.9)	2 (6.9)	

However, a significant difference was observed in maternal heart rates between the two groups. There was a notably higher prevalence of maternal heart rates above 100 beats per minute in untreated patients compared to those on beta-blockers (16% vs 2.6%, respectively), *P*<.01. This result aligns with the known bradycardic effect of beta-blockers.

When the analysis focused on the subgroup of patients treated with bisoprolol, fetal heart rates were not significantly different between the groups. Specifically, 100% of fetuses from patients on bisoprolol had heart rates between 110 and 150 bpm, compared to 93% of fetuses from untreated patients (*P*=1).

The secondary analysis on obstetric and neonatal outcomes revealed several significant differences between the groups, as shown in Table 4. Significant differences were noted in the onset of labor and mode of delivery. Specifically, cesarean sections were more frequent in patients on beta-blockers (53%) compared to untreated patients (8.9%), *P*<.01. This difference predominantly concerns cesarean deliveries before labor, linked to the underlying chronic pathology of these patients, leading to specific labor management and a risk of cesarean section due to cardiac decompensation.

**Table 4:** Secondary Analysis. Obstetric and neonatal outcome among untreated patients and patients with beta-blockers

Colonne1	Untreated patients *n*=45 (%)	Patients with beta-blockers *n*=45 (%)	*P*
Gestational age SA, median (IQR)	38 (36.6–38.4)	38 (36.6–38.4)	1
Onset of labor			
Spontaneous	27 (60)	7 (16)	<.01
Induction	14 (31)	14 (31)	
C-section	4 (8.9)	24 (53)	
Mode of delivery			
Spontaneous vaginal	28 (62)	13 (29)	<.01
Instrumental vaginal	6 (13)	5 (11)	
C-section	11 (24)	27 (60)	
Birth weight g, median (IQR)	2890 (2575–3310)	2625 (2260–3070)	<.01
Birth percentile <10th	6 (13)	16 (36)	.014

Regarding birth weight, the results suggest that newborns from mothers on beta-blockers tend to have a lower birth weight than those from untreated mothers, *P*<.01. These findings suggest a potential association between beta-blocker use during pregnancy and an increased risk of neonatal hypotrophy.

## Discussion

### Principal findings

Our study revealed a differential effect of beta-blockers on maternal and fetal heart rates in patients treated with beta-blockers during pregnancy compared to untreated pregnant women.

### Results in the context of what is known

The literature is limited concerning the hemodynamic effects of beta-blockers on fetuses. Our work contributes uniquely to this area by focusing specifically on the impact of beta-blockers on mean fetal heart rate.

Our findings complement existing research on labetalol (alpha-beta-blocker treatment), which, due to its use in hypertensive disorders of pregnancy, has a richer literature. For instance, a systematic review published in 2004 gathered studies on the impact of major antihypertensive drugs on fetal heart rate during pregnancy.^18^ The review included studies on labetalol, methyldopa, nifedipine, and hydralazine primarily. The authors did not find adverse effects of these treatments on fetal heart rate. In the neonatal period, the effects of Labetalol have been described, by Thewissen et al^19^ who found no differences in newborns of mothers treated with Labetalol compared to the control group concerning heart rate, blood pressure, and the need for vasopressor support.

Regarding the molecules studied in our research, Montan et al's^20^ 1984 trial compared the average fetal heart rate in 40 full-term pregnant women before and after initiating atenolol treatment for hypertension. They found a statistically significant decrease in the average fetal heart rate: 143 vs 133 (*P*<.001) after atenolol initiation. However, the clinical impact of such a decrease appears minimal, as the fetal heart rate remains within normal limits, not raising concerns during monitoring.

Montan et al also observed a decrease in variability of 5 to 10 bpm for at least 20 minutes in 13.1% of fetuses after atenolol introduction compared to 2.3% before treatment (*P*<.01). This parameter could falsely alert during monitoring, especially among fetuses suspected of being hypotrophic in patients treated with beta-blockers. Our study did not investigate this parameter, limiting the applicability of our findings. A more comprehensive or automated approach to interpreting fetal heart rate in patients on beta-blockers could offer more precise information for better monitoring interpretation in these high-risk obstetric patients.

Among patients with Marfan syndrome, two were treated with nebivolol. There is no data on the specific effect of this molecule on fetal hemodynamic parameters. A study in rats published in 2016 by Altoama et al^21^ compared the effects of bisoprolol and nebivolol on fetuses and found a decrease in average fetal weight gain when the mother was treated with nebivolol (*P*<.01). The effects on heart rate were not studied.

## Clinical implication

This result provides guidance to clinicians in interpreting fetal cardiac monitoring in these patients with chronic conditions, often at high obstetric risk.

More broadly, patients on beta-blocker treatment during pregnancy require close monitoring and appropriate clinical management to minimize risks to both the mother and the fetus. Further studies are needed to validate our findings and understand the underlying pathophysiological mechanisms.

## Research implications

From a pathophysiological perspective, it is established that so-called “cardio-selective” beta-blockers inhibiting beta-1 receptors like bisoprolol and atenolol cross the placental barrier.^22^ These molecules could, therefore, have a potential negative chronotropic or inotropic effect on the fetus. One hypothesis we can propose to explain the absence of effects of these molecules is the immaturity of the fetus's beta-adrenergic receptors. Further research is needed to validate this hypothesis.

Contradictory results exist in animals, notably in the 2022 article by Khandoker et al.^11^ In this work, conducted in mice, subcutaneous injection of propranolol in pregnant females led to a decrease in fetal heart rate compared to saline injection, with no effect on maternal heart rate. Given that the transplacental passage of beta-blockers in mice has not been demonstrated,^23^ the extrapolation of animal models to human pregnancy remains limited.

## Strengths and limitations

It is important to note that this study has certain inherent limitations due to its design. It is a retrospective, bicentric case-control study, which potentially limits the external validity of our results. Furthermore, the retrospective nature of the study could introduce selection and data collection biases. However, thanks to the computerized collection of our data, the comprehensive nature of the data is ensured.

Additionally, the sample sizes per treatment were small due to the rarity of the involved pathologies, which may limit the statistical power of the analysis and make it difficult to detect significant differences. Larger sample sizes could allow subgroup analyses and more detailed results per molecule. A potential dose-dependent effect could also be studied in a larger cohort.

Despite these limitations, this study has several strengths worth noting. The specificity of recruitment of maternal pathologies from the two maternity involved in this study provides genuine originality to our results. The matching between groups on important variables such as age, parity, and gestational age strengthens the robustness of the obtained results. The observed difference in heart rate in mothers on beta-blockers compared to the control group supports good treatment adherence by patients, reinforcing our findings. Similarly, the significant increase in neonatal hypotrophy in treated patients, a known effect of beta-blockers,^6^ aligns with fetal exposure to these treatments.

Finally, excluding patients with pathologies that could interfere with fetal heart rate constitutes a rigorous methodological approach, allowing for better isolation of the beta-blockers’ effect on fetal heart rate.

## Conclusion

Our study did not find a significant effect of beta-blockers on fetal heart rate in mothers treated during pregnancy compared to the control group. These women require specialized care management, and the interpretation of fetal monitoring should not be biased by maternal treatment. Further research is needed to understand the underlying physiological mechanisms.

## CRediT authorship contribution statement

**Sarah Hautier:** Writing – original draft, Investigation, Funding acquisition, Formal analysis, Data curation. **Thi Minh Thu Nguyen:** Resources. **Arane Kim:** Resources. **Tiphaine Barral:** Resources. **Dominique Luton:** Validation, Supervision, Conceptualization.
